# A proposal for further developing fatigue-related post COVID-19 health states for burden of disease studies

**DOI:** 10.1186/s13690-023-01212-1

**Published:** 2023-11-02

**Authors:** Grant M. A. Wyper, Scott A. McDonald, Juanita A. Haagsma, Brecht Devleesschauwer, Periklis Charalampous, Rishma Maini, Pierre Smith, Sara M. Pires

**Affiliations:** 1 Place and Wellbeing Directorate, Public Health Scotland, Glasgow, United Kingdom; 2 School of Health and Wellbeing, University of Glasgow, Glasgow, United Kingdom; 3 Centre for Infectious Disease Control, National Institute for Public Health and the Environment, Bilthoven, The Netherlands; 4 Erasmus MC University Medical Center, Rotterdam, The Netherlands; 5 Department of Epidemiology and Public Health, Sciensano, Brussels, Belgium; 6 Department of Translational Physiology, Infectiology and Public Health, Ghent University, Merelbeke, Belgium; 7 Clinical and Protecting Health Directorate, Public Health Scotland, Edinburgh, United Kingdom; 8 Public Health Department, NHS Fife, Fife, United Kingdom; 9 Institute of Health and Society (IRSS), Université catholique de Louvain, Brussels, Belgium; 10 National Food Institute, Technical University of Denmark, Lyngby, Denmark

**Keywords:** Long COVID, Post COVID-19 condition, Disability-adjusted life year, Years lived with disability, Fatigue, Disability weight, European burden of disease network

## Abstract

Previous efforts to estimate the burden of fatigue-related symptoms due to long COVID have a very high threshold for inclusion of cases, relative to the proposed definition from the World Health Organization. In practice this means that milder cases, that may be occurring very frequently, are not included in estimates of the burden of long COVID which will result in underestimation. A more comprehensive approach to modelling the disease burden from long COVID, in relation to fatigue, can ensure that we do not only focus on what is easiest to measure; which risks losing focus of less severe health states that may be more difficult to measure but are occurring very frequently. Our proposed approach provides a means to better understand the scale of challenge from long COVID, for consideration when preventative and mitigative action is being planned.

## Background

Post COVID-19 condition, more commonly known as long COVID, refers to a wide and diverse constellation of symptoms occurring following the acute phase of COVID-19 infection [1, 2]. Population-level burden of disease studies on COVID-19 that have incorporated long COVID indicate that health loss from long COVID contributed more to estimates of YLD (Years Lived with Disability) than health loss from the acute phase of infection. However, published estimates contain a large degree of uncertainty due to inconsistencies in definitions of long COVID across studies [3]. These uncertainties were caused by clear difficulties in establishing estimates of: the risk of developing, incidence, and duration, of an exhaustive list of long COVID symptoms. Another limiting factor was the inability to match any epidemiological inputs to corresponding disability weights at a level that differentiated by severity.

## Initial approaches to estimating the burden of COVID-19

In late summer of 2020, members of the European Burden of Disease Network convened to develop a consensus approach for estimating disability-adjusted life years (DALYs) due to COVID-19. DALYs combine time lost due to morbidity, using YLD, and time lost due to premature death using Years of Life Lost [4–6]. Particular considerations were given to data availability and how the quality of epidemiological data may have varied by country, to ensure that the model not only had a theoretical basis but was also applicable in different settings.

Although reports on the occurrence of long COVID had emerged when the European Burden of Disease Network’s consensus model was developed, epidemiological data were sparse. Given the gaps in knowledge at the time, the European Burden of Disease Network’s consensus models conservative recommendation was to include a single health state for long COVID. This involved using the disability weight for ‘infection, post-acute consequences’ to estimate YLD. This was similar to the approach the Global Burden of Disease (GBD) study have previously used to model the post-acute consequences of dengue and ebola [7]. The disability weight for this health state is a relatively high value of 0.219 [8]. Additionally, the value was fixed meaning that there was no scaling of degree of symptoms, for example from mild to severe. This presents a limiting factor when trying to match case definitions to the health state.

Although the mechanisms underlying the pathophysiology of long COVID are still debated, various hypotheses have been put forward, each of which can induce symptoms with different degrees of severity. On the one hand, long COVID may be related to organ damage following acute COVID-19 infection such as myocardial infection or renal failure, leading to persistent symptoms [9]. Another hypothesis is it is related to a prolonged pro-inflammatory response related to SARS-CoV-2, inducing an atypical response of the immune system and mast cells, and persistent symptoms [10]. In relation to the first hypothesis, this presents additional uncertainties in outcome-based YLD estimates as symptoms could plausibly present as cases of non-communicable disease, such as through increases in the prevalence of cardiovascular conditions, which could lead to double-counting in comprehensive burden of disease studies. Finally, the symptomatology of long COVID has been compared to that of fibromyalgia [11]. However, disability weights are not available for the latter as it is not included in the GBD study.

## Evolution of approaches to measuring long COVID

More recently, the GBD Long COVID Collaborators published estimates of the non-fatal disease burden of long COVID [12]. This provided a welcome development from focusing solely on the fatigue-related post-acute consequences, and assessed the disease burden from three clusters of long COVID symptoms: persistent fatigue with bodily pain or mood swings; cognitive problems; and ongoing respiratory problems. Whilst the GBD Long COVID Collaborators have incorporated additional neurological and respiratory disease clusters according to severity, further developments could be considered for the fatigue-related disease cluster as their approach is still in-line with the model proposed from the European Burden of Disease Network over applying the disability weight of 0.219 for ‘infection, post-acute consequences’ to cases with persistent fatigue with bodily pain or mood swings.

The clinical definition of long COVID proposed by the World Health Organization in relation to fatigue is not as restrictive as the one defined by the European Burden of Disease Network or GBD Long COVID Collaborators [12]. In practice this means that a proportion of patients suffering fatigue-related health states – but at a lower level of severity – do not meet the strict threshold to contribute to YLD calculations, thus potentially underestimating the true burden of fatigue-related long COVID. In addition, failing to capture less severe instances of fatigue may lead to studies applying a single disability weight, that is too severe, to cases that span a range of severity states. These limitations are all the more important as several studies have highlighted fatigue as the most prevalent symptom of long COVID [13–15]. Neither of these approaches are preferable, but both can be understood, as researchers attempt to understand the impact of long COVID on population health loss.

## Proposal for developing fatigue-related health states

The aforementioned issues could be circumvented by extending the range of potential disability weights available for fatigue-related health states. We suggest that the GBD study disability weights used for mild, moderate, and severe anemia could be used to define health states less debilitating than the post-acute consequences of an infectious disease to further augment estimates of the long COVID fatigue disease cluster (Table 1). Several conditions have psychological and pathological symptoms and frequently both these symptoms are included in the health state description (e.g. paraplegia, where feelings of depression are also included in the lay description). For infectious disease, post-acute consequences (fatigue, emotional lability, insomnia) this psychological component is also included. Of course, for some patients the severity of the psychological component may differ, but this is true for many other conditions with a psychological component. Ideally, we would have disability weights for a wide spectrum of severity and diversity of clinical manifestations of a disease; however, in reality available epidemiological data is often limited in terms of information on severity and diversity of clinical manifestations, and we have to make cruder groupings in terms of clinical manifestation of a disease.

These health states link to the GBD-defined symptom cluster of persistent fatigue with bodily pain or mood swings. Importantly, they are on a multiple scale of increasing severity allowing for more accurate matching to epidemiological estimates to reflect that certain symptoms are not only more debilitating, but may have important differences in incidence and duration.

**Table 1:** Proposed disability weights for long COVID fatigue-related health states

Health state	Health state description	Disability weight (95% uncertainty interval)
Anemia, mild	feels slightly tired and weak at times, but this does not interfere with normal daily activities.	0.004 (0.001–0.008)
Anemia, moderate	feels moderate fatigue, weakness, and shortness of breath after exercise, making daily activities more difficult.	0.052 (0.034–0.076)
Anemia, severe	feels very weak, tired and short of breath, and has problems with activities that require physical effort or deep concentration.	0.149 (0.101–0.209)
Infectious disease, post-acute consequences (fatigue, emotional lability, insomnia)	is always tired and easily upset. The person feels pain all over the body and is depressed.	0.219 (0.148–0.308)

Further research could be undertaken to test the validity of this proposal. Long COVID patients with fatigue could be surveyed over how closely their symptoms match to the health state descriptions outlined in Table 1.

## Conclusion

A more comprehensive approach to modelling long COVID in relation to fatigue can ensure that we do not only focus on what is easiest to measure, and thus lose focus of less severe health states that may be more difficult to measure but are occurring very frequently. The approach which we have outlined will help researchers more accurately, and comprehensively, estimate the burden from long COVID. This will provide a better understanding of the scale of challenge from long COVID, for consideration when preventative and mitigative action is being planned.

## Data Availability

Not applicable.

## Abbreviations

COVID-19: Coronavirus Disease 2019
DALYs: Disability-Adjusted Life Years
GBD: Global Burden of Disease
YLD: Years Lived with Disability
